# Effect of biostimulants on the growth, yield and nutritional value of *Capsicum annuum* grown in an unheated plastic tunnel

**DOI:** 10.1038/s41598-021-01834-x

**Published:** 2021-11-16

**Authors:** Joanna Majkowska-Gadomska, Artur Dobrowolski, Krzysztof K. Jadwisieńczak, Zdzisław Kaliniewicz, Anna Francke

**Affiliations:** 1 Department of Agroecosystems and Horticulture, Faculty of Agriculture and Forestry, University of Warmia and Mazury in Olsztyn, Plac Łódzki 3, 10–718, Olsztyn, Poland; 2 Department of Heavy Duty Machines and Research Methodology, University of Warmia and Mazury in Olsztyn, Oczapowskiego 11, 10–719, Olsztyn, Poland

**Keywords:** Natural products, Agroecology, Mechanical engineering

## Abstract

Recent years have witnessed an increased interest in the cultivation and consumption of peppers. Therefore, new solutions are being sought to provide pepper plants with the most favorable conditions for growth and development. In view of the above, the aim of this study was to evaluate the effect of selected biostimulants on the biometric parameters, yield and nutritional value of *Capsicum annuum* fruit. The research hypothesis postulates that biostimulants can increase the yield and improve the nutritional quality of pepper fruit. The experiment was conducted in an unheated plastic tunnel. The experimental materials comprised three sweet (‘Solario F_1_’, ‘Turbine F_1_’ and ‘Whitney F_1_’) and two hot (‘Cyklon’ and ‘Palivec’) cultivars of *C. annuum*. It was found that the combined application of environmentally-friendly microbial-based biostimulants (BB Soil, BB Foliar, Multical, MK5 and Biocin F) did not clearly improve the morphological traits of pepper fruit, yield or the concentrations of sugars and organic acids in fruit, therefore their use is not economically justified. Hot peppers had a higher content of dry matter, total sugars and L-ascorbic acid than sweet peppers. The analyzed biostimulants increased nitrate (V) concentrations in the fruit of both hot and sweet peppers. ‘Turbine F_1_’ and ‘Solario F_1_’ were particularly prone to nitrate (V) accumulation in fruit, therefore the use of biostimulants should be limited in their cultivation. Pepper fruits with the largest horizontal diameter and the thickest skin should be preferred because these traits are associated with high sugar content.

## Introduction

*Capsicum annum* L. is a thermophilous species. It has a long growing season and high environmental, soil and light requirements. Recent years have witnessed an increased interest in the cultivation and consumption of peppers, both in Poland and around the world^1–3^. The selection of high-yielding cultivars that are resistant to pathogens and adverse growing conditions is one of the key factors in integrated vegetable production. Cultivars that are more tolerant of physiological disorders are able to survive under stress conditions^4^. Different cultivars are also selected to meet specific market demands^5,6^. Rising consumer expectations regarding the quality of vegetables and increased consumer awareness of the problem of global food security have promoted producers to constantly improve the functional properties of foods. An increase in the quality of the edible parts of vegetable plants should be accompanied by maximizing yields per unit area while minimizing environmental impacts^7,8^. In order to meet this goal, ecologically sustainable and economically viable products should be used in crop production^9^. In the era of global climate change, when weather conditions may negatively affect crop yields and quality, modern agriculture has to rely on the use of not only fungicides, herbicides and insecticides but also numerous products classified as plant growth regulators or biostimulants^10^. Research has shown that biostimulants have a beneficial influence on plant performance by enhancing their resistance to diseases and pests, and increasing yields^11–13^. Biostimulants influence metabolic and enzymatic processes in plants, thus increasing their yield and quality. According to Du Jardin^14^, a plant biostimulant is any substance or microorganism applied to plants, seeds or in the rhizosphere with the aim to stimulate natural processes in plants, enhance nutrition efficiency and/or abiotic stress tolerance, regardless of its nutrient content, or a mixture of such substances and/or microorganisms. Zarzecka et al.^15^ demonstrated that mechanical and chemical treatments combined with biostimulants were profitable in the cultivation of edible potatoes. The application of biostimulants and herbicides decreased the cost of cultivation measures and increased economic efficiency, compared with mechanical treatments alone. Similar results were reported by Kocira^16^ for soybeans, Nowosad et al.^17^ for beans, Budzyński et al.^18^ for winter oilseed rape, Bonini et al.^19^ for sweet peppers and Cirillo et al.^20^ for grapevines.

The introduction of new microbial-based biostimulants to the market has promoted the authors to undertake the present study. These products have recently gained increasing interest because they promote microbiological soil life, contribute to faster heating of the soil in spring, and increase the water retention capacity of soil. They contain microbial solutions consisting mainly of lactic acid bacteria, photosynthetic bacteria and yeasts, and they do not contain genetically modified microorganisms^21,22^.

The research hypothesis postulates that the application of BB Soil, BB Foliar, Multical, MK5 and Biocin F biostimulants in the cultivation of *C. annuum* can improve the biometric parameters, yield and nutritional value (content of sugars, organic acids and nitrates (V)) of fruit.

The aim of this study was to evaluate the effect of a selected group of biostimulants on the biometric parameters, yield and nutritional value of *C. annuum* fruit, and to determine the correlations between these traits under the climatic conditions of north-eastern Poland where pepper cultivation is difficult.

## Results and discussion

### Plant and fruit characteristics

#### Biometric parameters of plants

The analyzed cultivars (characterized by desirable morphological, physical and chemical properties, uniform ripening, suitability for mechanical harvesting, high productivity, resistance to diseases and pests) and the application of modern farming technologies can have significant effects on crop yields and quality and, in consequence, production profitability, which was also observed by other authors^11,23–25^.

The influence of the combined application of biostimulants on the biometric parameters of plants in the analyzed cultivars of *C. annuum* is presented in Table 1. The analyzed biostimulants had no significant effect on the values of leaf greenness (SPAD), relative to the control treatment. In the tested cultivars, the mean values of this parameter ranged from 52.5 (cv. ‘Turbine F_1_’) to 58.8 (cv. ‘Cyklon’). In general, leaf greenness was not significantly affected by the treatment × cultivar interaction, although two homogeneous groups were identified: cvs. ‘Turbine F_1_’ and ‘Palivec’, and cvs. ‘Solario F_1_’, ‘Whitney F_1_’ and ‘Cyklon’.

**Table 1:** Effect of the combined application of biostimulants on the biometric parameters (mean values ± standard deviations) of plants in the analyzed cultivars of *Capsicum*.

Factor	Source of variation	*L*_*g*_ [−]	*m*_*p*_ [g]	*m*_*r*_ [g]
Treatment (A)	C	55.3 ± 3.3 a	169 ± 47 a	185 ± 79 a
	I	55.3 ± 3.0 a	211 ± 59 a	195 ± 62 a
	II	55.0 ± 3.7 a	191 ± 65 a	171 ± 54 a
	III	55.8 ± 4.6 a	174 ± 70 a	176 ± 63 a
	*p*-value	0.943	0.226	0.775
Cultivar (B)	Solario F_1_ (S)	56.6 ± 2.9 a	248 ± 52 a	196 ± 69 a
	Whitney F_1_ (W)	56.1 ± 2.3 a	229 ± 40 ab	214 ± 50 a
	Turbine F_1_ (T)	52.5 ± 3.2 b	150 ± 21 c	167 ± 40 ab
	Cyklon (K)	58.8 ± 2.5 a	193 ± 36 b	220 ± 49 a
	Palivec (P)	52.8 ± 2.4 b	112 ± 27 c	112 ± 47 b
	*P*-value	< 0.001	< 0.001	< 0.001
A × B	C × S	55.0 ± 2.4 ab	177 ± 22 c-f	153 ± 19 e–g
	C × W	55.2 ± 2.8 ab	238 ± 26 a-d	289 ± 27 ab
	C × T	54.4 ± 2.9 ab	172 ± 22 e–g	218 ± 19 cd
	C × K	59.7 ± 2.5 a	149 ± 13 f.-i	202 ± 15 c-e
	C × P	52.4 ± 2.4 ab	108 ± 11 g-i	64 ± 9 i
	I × S	55.9 ± 3.1 ab	286 ± 30 a	118 ± 15 f.–h
	I × W	56.9 ± 3.1 ab	248 ± 24 ab	198 ± 13 c-e
	I × T	54.5 ± 1.6 ab	148 ± 13 f.-i	175 ± 12 de
	I × K	57.4 ± 2.3 ab	225 ± 21 a-e	298 ± 26 a
	I × P	51.7 ± 1.7 ab	151 ± 8 f.–h	184 ± 16 de
	II × S	56.5 ± 2.7 ab	242 ± 19 a-c	242 ± 23 bc
	II × W	56.1 ± 2.6 ab	253 ± 36 ab	203 ± 19 c-e
	II × T	51.5 ± 2.7 ab	137 ± 15 f.-i	116 ± 10 f.-i
	II × K	58.9 ± 3.1 a	220 ± 22 b-e	186 ± 14 de
	II × P	52.0 ± 2.0 ab	102 ± 13 hi	111 ± 10 g-i
	III × S	59.1 ± 3.3 a	287 ± 33 a	273 ± 32 ab
	III × W	56.3 ± 3.4 ab	175 ± 20 d-f	165 ± 13 d-f
	III × T	49.4 ± 3.2 b	142 ± 19 f.-i	160 ± 10 e–g
	III × K	59.3 ± 3.0 a	179 ± 17 c-f	195 ± 11 c-e
	III × P	55.0 ± 2.9 ab	86 ± 8 i	89 ± 8 hi
	*p*-value	0.273	< 0.001	< 0.001

Means followed by different letters are significantly different for each parameter (the level of significance, the *p*-value, is shown in the table).

*L*_*g*_-leaf greenness (SPAD), *m*_*p*_-weight of aboveground plant parts, *m*_*r*_-root weight.

C-control treatment; I-BB Foliar + Multical; II-BB Foliar + Multical + MK5; III-BB Foliar + Multical + MK5 + Biocin F.

The average weight of aboveground plant parts ranged from 112 g (cv. ‘Palivec’) to 248 g (cv. ‘Solario F_1_’), and average root weight ranged from 112 g (cv. ‘Palivec’) to 220 g (cv. ‘Cyklon’). These parameters were not significantly affected by the method of biostimulant application, but their values were highest in treatment I (combined application of BB Soil, BB Foliar and Multical). The analyzed *C. annuum* cultivars can be divided into three homogeneous groups based on the weight of aboveground plant parts, and into two homogeneous groups based on root weight, but the biostimulants exerted different effects on these parameters in each cultivar. The weight of aboveground plant parts was highest in cv. ‘Solario F_1_’ in treatments I and III, and lowest in cv. ‘Palivec’ in treatment III. Root weight was highest in cv. ‘Cyklon’ in treatment I, and lowest in cv. ‘Palivec’ in the control treatment.

It can be concluded that the combined application of the tested biostimulants had a minor effect on the biometric parameters of pepper plants. In contrast, Thevanathan et al.^26^ and Bai et al.^27^ demonstrated that algal extracts had a considerable influence on plant height (35% increase) in pulses. Bilal^28^, Abou-Shlell et al.^29^ and Hamed^30^ found that the natural foliar nano-fertilizer Lithovit positively affected the vegetative growth of crop plants.

#### Yield

The fruit yields of the analyzed *C. annuum* cultivars treated with biostimulants applied in different combinations are presented in Table 2. Similarly to the values of leaf greenness and biometric parameters of plants, early, marketable and total yields were determined mostly by varietal traits, whereas biostimulants exerted a minor effect. On average, ‘Whitney F_1_’ was the highest-yielding cultivar, and ‘Cyklon’ was the lowest-yielding cultivar. Sweet cultivars were characterized by higher yields than hot cultivars, and the best results were noted in treatment II (combined application of BB Soil, BB Foliar, Multical and MK5), although no significant differences were observed relative to the control treatment and the remaining experimental treatments. The early yield ranged from 0.2 kg·m^−2^ (cv. ‘Cyklon’) to 3.8 kg·m^−2^ (cv. ‘Whitney F_1_’), and ‘Cyklon’ and ‘Palivec’ (hot cultivars) were characterized by similar early yields. The marketable yield was lowest in cv. ‘Cyklon’ (3.1 kg·m^−2^) and highest in cv. ‘Turbine F_1_’ (7.3 kg·m^−2^). ‘Turbine F_1_’ and ‘Whitney F_1_’ were characterized by comparable marketable yields. Similar effects were observed with regard to total yield. An analysis of the values of marketable and total yields revealed that the percentage of marketable fruits was higher in hot cultivars (approx. 100% on average) than in sweet cultivars (approx. 93–99% on average), and it was lowest in cv. ‘Whitney F_1_’ in treatment II (combined application of BB Soil, BB Foliar, Multical and MK5)—around 88%. The analyzed *C. annuum* cultivars responded differently to the tested combinations of biostimulants in terms of yield, but they did not differ significantly in total fruit yield, although nine homogeneous groups were identified.

**Table 2:** Effect of the combined application of biostimulants on fruit yield (mean values ± standard deviations) in the analyzed cultivars of *Capsicum annuum*.

Factor	Source of variation	*Y*_*e*_ [kg m^−2^]	*Y*_*m*_ [kg m^−2^]	*Y*_*t*_ [kg m^−2^]
Treatment (A)	C	1.5 ± 1.1 a	5.5 ± 1.8 a	5.7 ± 1.9 a
	I	1.8 ± 1.3 a	5.4 ± 1.3 a	5.5 ± 1.5 a
	II	1.8 ± 1.5 a	6.3 ± 2.0 a	6.6 ± 2.2 a
	III	1.5 ± 1.5 a	5.4 ± 1.6 a	5.5 ± 1.7 a
	*p*-value	0.884	0.383	0.312
Cultivar (B)	Solario F_1_ (F)	1.4 ± 0.7 c	5.9 ± 0.7 b	6.3 ± 0.9 b
	Whitney F_1_ (W)	3.8 ± 0.5 a	7.0 ± 0.7 a	7.5 ± 1.0 a
	Turbine F_1_ (T)	2.2 ± 0.4 b	7.3 ± 1.1 a	7.3 ± 1.2 a
	Cyklon (K)	0.2 ± 0.1 d	3.1 ± 0.5 d	3.1 ± 0.6 d
	Palivec (P)	0.5 ± 0.1 d	5.0 ± 0.4 c	5.0 ± 0.6 c
	*P*-value	< 0.001	< 0.001	< 0.001
A × B	C × S	1.7 ± 0.1 ef	5.8 ± 0.7 c-f	6.5 ± 1.0 b-f
	C × W	3.3 ± 0.4 bc	6.9 ± 0.6 b-d	7.1 ± 0.8 a-d
	C × T	1.7 ± 0.2 ef	7.4 ± 0.5 ab	7.5 ± 0.7 a-c
	C × K	0.2 ± 0.1 g	2.7 ± 0.2 i	2.7 ± 0.4 hi
	C × P	0.5 ± 0.1 g	4.8 ± 0.3 f.–h	4.8 ± 0.8 e–h
	I × S	2.2 ± 0.1 de	5.8 ± 0.5 c-f	5.9 ± 0.7 c-f
	I × W	3.7 ± 0.4 ab	7.0 ± 0.8 bc	7.4 ± 1.0 a-c
	I × T	2.2 ± 0.2 de	6.1 ± 0.4 b-f	6.2 ± 0.6 c-f
	I × K	0.3 ± 0.1 g	3.6 ± 0.2 g-i	3.6 ± 0.5 g-i
	I × P	0.5 ± 0.1 g	4.6 ± 0.2 f.–h	4.6 ± 0.4 f.-i
	II × S	1.3 ± 0.1 f.	6.7 ± 0.7 b-d	7.2 ± 0.6 a-d
	II × W	4.2 ± 0.4 a	7.5 ± 0.7 ab	8.5 ± 0.9 ab
	II × T	2.6 ± 0.4 cd	8.8 ± 0.7 a	8.8 ± 1.1 a
	II × K	0.3 ± 0.1 g	3.5 ± 0.3 hi	3.5 ± 0.3 g-i
	II × P	0.5 ± 0.1 g	5.1 ± 0.3 e–g	5.1 ± 0.6 d-g
	III × S	0.5 ± 0.1 g	5.5 ± 0.5 d-f	5.5 ± 0.5 c-g
	III × W	4.1 ± 0.3 a	6.5 ± 0.5 b-e	7.0 ± 0.8 a-e
	III × T	2.1 ± 0.2 de	6.8 ± 0.5 b-d	6.8 ± 0.9 a-f
	III × K	0.2 ± 0.1 g	2.6 ± 0.2 i	2.6 ± 0.4 i
	III × P	0.6 ± 0.1 g	5.5 ± 0.3 d-f	5.5 ± 0.4 c-g
	*p*-value	< 0.001	< 0.001	0.055

Means followed by different letters are significantly different for each parameter (the level of significance, the *p*-value, is shown in the table).

*Y*_*e*_-early yield, *Y*_*m*_-marketable yield, *Y*_*t*_-total yield.

C-control treatment; I-BB Foliar + Multical; II-BB Foliar + Multical + MK5; III-BB Foliar + Multical + MK5 + Biocin F.

A positive effect of titanium application on crop yields was also observed by Marcinek and Hetman^31^ in Sparaxis tricolor Ker Gawl, and by Grajkowski and Ochmian^32^ in raspberries. In a study of strawberries conducted by Michalski^33^, the effectiveness of titanium in plant nutrition varied across years. Dobromilska^34^ reported that the foliar application of titanium contributed to an increase in tomato yields and significantly enhanced the vegetative growth of tomato plants, including an increase in plant height, stem diameter and the number of leaves per plant. Normal vegetative growth and development contributes to improving crop quality, and genetic factors play a major role under identical growing conditions^6^.

#### Biometric parameters of fruit

The biometric parameters of fruit in the analyzed *C. annuum* cultivars are presented in Table 3. Similarly to the previously described traits, the biometric parameters of pepper fruit were not significantly influenced by the tested biostimulants. The biometric parameters of fruits were affected by varietal traits, and differences were noted between sweet and hot cultivars. The fruits of sweet cultivars had higher weight, larger horizontal diameter, thicker skin and smaller vertical diameter, compared with hot cultivars. No significant treatment × cultivar interaction was found for the weight, vertical diameter or horizontal diameter of fruit, although several homogeneous groups could be identified based on the differences between cultivars.

**Table 3:** Effect of the combined application of biostimulants on the biometric parameters (mean values ± standard deviations) of fruit in the analyzed cultivars of *Capsicum annuum*.

Factor	Source of variation	*m*_*f*_ [g]	*d*_1_ [cm]	*d*_2_ [cm]	*s* [mm]
Treatment (A)	C	103 ± 66 a	10.5 ± 2.4 a	5.4 ± 2.5 a	5.0 ± 1.7 a
	I	107 ± 83 a	10.7 ± 2.3 a	5.6 ± 2.4 a	4.8 ± 1.8 a
	II	108 ± 73 a	10.7 ± 2.5 a	5.6 ± 2.8 a	4.6 ± 1.6 a
	III	102 ± 68 a	11.0 ± 2.0 a	5.6 ± 2.5 a	5.0 ± 1.7 a
	*P*-value	0.995	0.944	0.994	0.926
Cultivar (B)	Solario F_1_ (S)	224 ± 27 a	9.5 ± 0.9 c	9.0 ± 0.8 a	6.1 ± 0.8 a
	Whitney F_1_ (W)	86 ± 19 c	9.4 ± 0.8 c	5.6 ± 0.6 c	6.4 ± 1.1 a
	Turbine F_1_ (T)	132 ± 22 b	9.1 ± 1.1 c	7.3 ± 0.7 b	5.7 ± 0.9 a
	Cyklon (K)	39 ± 4 d	11.0 ± 0.8 b	3.5 ± 0.6 d	3.3 ± 0.4 b
	Palivec (P)	46 ± 3 d	14.6 ± 0.9 a	2.4 ± 0.2 e	2.9 ± 0.4 b
	*P*-value	< 0.001	< 0.001	< 0.001	< 0.001
A × B	C × S	212 ± 22 a	9.7 ± 0.9 c-e	8.6 ± 0.7 a-d	6.7 ± 0.3 a
	C × W	103 ± 19 b-d	8.8 ± 1.1 de	5.6 ± 0.6 e–g	5.7 ± 0.7 ab
	C × T	117 ± 22 b-d	8.0 ± 0.9 e	7.4 ± 0.9 b-e	6.4 ± 0.7 ab
	C × K	40 ± 5 e	11.6 ± 0.9 bc	3.3 ± 0.3 h	3.1 ± 0.2 de
	C × P	45 ± 4 e	14.2 ± 1.0 ab	2.2 ± 0.1 h	3.1 ± 0.3 de
	I × S	247 ± 33 a	8.9 ± 0.7 c-e	8.8 ± 0.9 a-c	6.7 ± 0.4 a
	I × W	79 ± 14 de	9.4 ± 0.7 c-e	5.6 ± 0.8 e–g	5.7 ± 1.0 ab
	I × T	134 ± 27 bc	9.4 ± 0.7 c-e	7.3 ± 0.6 c-f	6.0 ± 1.0 ab
	I × K	34 ± 4 e	11.2 ± 0.9 cd	4.0 ± 0.3 gh	3.3 ± 0.4 de
	I × P	44 ± 3 e	14.6 ± 0.7 a	2.4 ± 0.1 h	2.4 ± 0.2 e
	II × S	222 ± 32 a	9.2 ± 1.0 c-e	9.3 ± 0.9 a	5.5 ± 0.7 a-c
	II × W	83 ± 20 c-e	10.0 ± 0.8 c-e	5.6 ± 0.7 e–g	6.8 ± 1.2 a
	II × T	149 ± 17 b	8.8 ± 0.8 de	7.6 ± 0.6 a-d	4.6 ± 0.6 b-d
	II × K	40 ± 3 e	10.5 ± 0.7 c-e	2.9 ± 0.2 h	3.2 ± 0.5 de
	II × P	46 ± 3 e	15.1 ± 1.1 a	2.5 ± 0.2 h	3.1 ± 0.3 de
	III × S	215 ± 15 a	10.0 ± 1.1 c-e	9.3 ± 0.8 a	5.5 ± 0.9 a-c
	III × W	81 ± 20 c-e	9.4 ± 0.6 c-e	5.5 ± 0.7 fg	7.2 ± 0.8 a
	III × T	128 ± 15 b-d	10.2 ± 0.9 c-e	6.8 ± 0.7 d-f	5.7 ± 0.3 ab
	III × K	40 ± 3 e	10.8 ± 0.8 cd	3.9 ± 0.7 gh	3.6 ± 0.6 c-e
	III × P	47 ± 3 e	14.5 ± 0.9 a	2.5 ± 0.1 h	3.0 ± 0.5 de
	*P*-value	0.263	0.165	0.493	0.004

Means followed by different letters are significantly different for each parameter (the level of significance, the *p*-value, is shown in the table).

*m*_*f*_-weight, *d*_1_-vertical diameter, *d*_2_-horizontal diameter, *s*-skin thickness.

C-control treatment; I-BB Foliar + Multical; II-BB Foliar + Multical + MK5; III-BB Foliar + Multical + MK5 + Biocin F.

Average fruit weight varied widely across cultivars, from 39 g (cv. ‘Cyklon’) to 224 g (cv. ‘Solario F_1_’). Hot cultivars (‘Cyklon’ and ‘Palivec’) formed a homogeneous group based on fruit weight. The fruit weight in hot cultivars of *C. annuum* was similar to that reported by Islam et al.^24^. Sweet and hot pepper cultivars differ also in fruit shape. The fruits of hot cultivars are long and narrow, whereas the fruits of sweet cultivars have similar horizontal and vertical dimeters. Sweet cultivars are similar in terms of vertical diameter, and they differ mostly in average horizontal diameter. Fruits with the smallest mean vertical diameter (9.1 cm) were harvested from plants of cv. ‘Turbine F_1_’, and fruits with the largest mean vertical diameter (14.6 cm) were harvested from plants of cv. ‘Palivec’. Fruits with the smallest mean horizontal diameter (2.4 cm) were harvested from plants of cv. ‘Palivec’, and fruits with the largest mean horizontal diameter (9.0 cm) were harvested from plants of cv. ‘Solario F_1_’.

The fruits of sweet and hot *C. annuum* cultivars had pericarps of similar thickness. In hot cultivars, average skin thickness ranged from 2.9 mm (cv. ‘Palivec’) to 3.3 mm (cv. ‘Cyklon’), and in sweet cultivars—from 5.7 mm (cv.‘Turbine F_1_’) to 6.4 mm (cv. ‘Whitney F_1_’).

#### Chemical composition of fruit

The proximate chemical composition of fruit in the analyzed *C. annuum* cultivars is presented in Table 4. The effects exerted by biostimulants on most chemical properties of pepper fruit (excluding L-ascorbic acid content) varied across cultivars. The applied biostimulants led to both an increase and a decrease in the content of the analyzed components in the studied cultivars. No significant differences in the concentrations of dry matter, total sugars, reducing sugars or L-ascorbic acid in pepper fruit were found between treatments. In comparison with the control treatment, significant differences were noted only for nitrate (V) levels in treatment I. The combined application of biostimulants led to an increase in the nitrate (V) content of fruit, which was nearly two-fold higher in treatment I than in the control group. The fruits of sweet cultivars had a lower content of dry matter, total sugars and L-ascorbic acid than the fruits of hot cultivars.

**Table 4:** Effect of the combined application of biostimulants on the chemical composition (mean values ± standard deviations) of fruit in the analyzed cultivars of *Capsicum annuum*.

Factor	Source of variation	*c*_*dm*_ [%]	*c*_*ts*_ [g∙100 g^−1^ f.w.]	*c*_*rs*_ [g∙100 g^−1^ f.w.]	*c*_*L*_ [mg∙100 g^−1^ f.w.]	*c*_*N*_ [mg N-NO_3_ kg^−1^ f.w.]
Treatment (A)	C	8.1 ± 2.2 a	5.7 ± 1.7 a	3.6 ± 1.0 a	100 ± 8 a	134 ± 50 b
	I	9.7 ± 3.1 a	5.0 ± 2.4 a	3.0 ± 1.4 a	101 ± 8 a	252 ± 124 a
	II	8.8 ± 1.6 a	5.0 ± 1.8 a	3.1 ± 1.4 a	102 ± 8 a	184 ± 66 ab
	III	9.8 ± 3.7 a	5.7 ± 2.8 a	3.5 ± 0.8 a	101 ± 9 a	213 ± 114 ab
	*P*-value	0.318	0.678	0.470	0.883	0.010
Cultivar (B)	Solario F_1_ (S)	7.6 ± 0.7 b	4.6 ± 0.3 c	3.4 ± 0.5 b	97 ± 9 b	230 ± 144 ab
	Whitney F_1_ (W)	6.4 ± 0.8 b	3.2 ± 1.0 d	2.4 ± 0.6 c	97 ± 7 b	168 ± 83 ab
	Turbine F_1_ (T)	7.5 ± 0.8 b	3.7 ± 0.9 cd	2.4 ± 1.0 c	97 ± 6 b	259 ± 121 a
	Cyklon (K)	11.6 ± 2.3 a	8.4 ± 1.2 a	5.1 ± 0.5 a	107 ± 6 a	187 ± 36 ab
	Palivec (P)	12.3 ± 2.1 a	6.9 ± 0.8 b	3.1 ± 0.7 bc	107 ± 6 a	136 ± 33 b
	*P*-value	< 0.001	< 0.001	< 0.001	< 0.001	0.020
A × B	C × S	7.0 ± 0.4 f.–h	4.6 ± 0.2 d	3.4 ± 0.3 cd	95 ± 8 a	133 ± 15 e–g
	C × W	5.8 ± 0.4 h	4.3 ± 0.3 de	2.8 ± 0.2 c-e	96 ± 7 a	106 ± 10 fg
	C × T	6.9 ± 0.7 f.–h	4.6 ± 0.3 d	2.9 ± 0.4 c-e	96 ± 6 a	125 ± 18 e–g
	C × K	11.4 ± 1.0 bc	8.3 ± 0.4 b	5.4 ± 0.5 a	105 ± 7 a	138 ± 14 e–g
	C × P	9.6 ± 0.6 cd	6.8 ± 0.7 c	3.3 ± 0.3 c-e	107 ± 7 a	85 ± 7 g
	I × S	8.5 ± 0.3 d-f	4.5 ± 0.4 de	3.8 ± 0.5 bc	94 ± 9 a	461 ± 58 a
	I × W	6.1 ± 0.4 gh	2.2 ± 0.1 f.	1.6 ± 0.2 fg	96 ± 7 a	301 ± 28 b
	I × T	7.9 ± 0.4 d-g	3.1 ± 0.1 ef	2.2 ± 0.2 ef	101 ± 7 a	183 ± 17 c-e
	I × K	11.2 ± 0.8 c	7.7 ± 0.5 bc	5.2 ± 0.6 a	107 ± 7 a	159 ± 12 c-f
	I × P	14.6 ± 0.9 a	7.7 ± 0.8 bc	2.2 ± 0.3 ef	105 ± 7 a	158 ± 7 c-f
	II × S	7.6 ± 0.6 e–h	4.5 ± 0.3 de	2.9 ± 0.2 c-e	104 ± 10 a	175 ± 36 c-f
	II × W	7.7 ± 0.4 d-h	4.1 ± 0.4 de	2.9 ± 0.2 c-e	98 ± 9 a	149 ± 19 d-g
	II × T	8.1 ± 0.8 d-g	2.5 ± 0.2 f.	0.9 ± 0.1 g	95 ± 6 a	305 ± 22 b
	II × K	9.0 ± 0.6 de	7.5 ± 0.6 bc	5.2 ± 0.5 a	107 ± 6 a	151 ± 15 d-g
	II × P	11.5 ± 0.7 bc	6.3 ± 0.5 c	3.4 ± 0.4 cd	108 ± 7 a	142 ± 19 e–g
	III × S	7.5 ± 0.6 e–h	4.6 ± 0.4 d	3.4 ± 0.3 cd	96 ± 8 a	150 ± 29 d-g
	III × W	6.2 ± 0.3 gh	2.4 ± 0.3 f.	2.4 ± 0.3 d-f	96 ± 7 a	118 ± 11 e–g
	III × T	7.2 ± 0.7 e–h	4.5 ± 0.2 de	3.4 ± 0.3 cd	94 ± 6 a	422 ± 26 a
	III × K	14.7 ± 0.9 a	10.2 ± 0.9 a	4.8 ± 0.4 ab	111 ± 8 a	215 ± 18 cd
	III × P	13.3 ± 0.9 ab	6.9 ± 0.8 bc	3.6 ± 0.6 c	108 ± 6 a	159 ± 16 c-f
	*P*-value	< 0.001	< 0.001	< 0.001	0.880	< 0.001

Means followed by different letters are significantly different for each parameter (the level of significance, the *p*-value, is shown in the table).

*c*_*dm*_-dry matter content, *c*_*ts*_-total sugar content, *c*_*rs*_-reducing sugar content, *c*_*L*_ –L-ascorbic acid content, *c*_*N*_-nitrate (V) content.

C-control treatment; I-BB Foliar + Multical; II-BB Foliar + Multical + MK5; III-BB Foliar + Multical + MK5 + Biocin F.

Average dry matter content ranged from 6.4% (cv. ‘Whitney F_1_’) to 7.6% (cv. ‘Solario F_1_’) in sweet peppers, and from 11.6% (cv. ‘Cyklon’) to 12.3% (cv. ‘Cyklon’) in hot peppers. Sweet and hot cultivars of *C. annuum* formed separate homogeneous groups. The analyzed cultivars differed significantly in the total sugar content of fruit, which was lowest in cv. ‘Whitney F_1_’ and highest in cv. ‘Cyklon’. Average total sugar content ranged from 3.2 to 4.6 g∙100 g^−1^ fresh weight in sweet peppers, and from 6.9 to 8.4 g∙100 g^−1^ fresh weight in hot peppers. Cultivars ‘Whitney F_1_’, ‘Turbine F_1_’ and ‘Palivec’, and ‘Solario F_1_’ and ‘Palivec’ formed homogeneous groups based on the reducing sugar content of fruit, which ranged from 2.4 g∙100 g^−1^ fresh weight (cv. ‘Whitney F_1_’ and ‘Turbine F_1_’) to 5.1 g∙100 g^−1^ fresh weight (cv. ‘Cyklon’). Average L-ascorbic acid content 97 mg∙100 g^−1^ fresh weight in sweet peppers, and 107 mg∙100 g^−1^ fresh weight in hot peppers. Similarly to the dry matter content of fruit, separate homogeneous groups were formed by sweet and hot cultivars of *C. annuum*. The combined application of biostimulants caused an increase in the average nitrate (V) content of pepper fruit, which ranged from 136 mg N-NO_3_ kg^−1^ fresh weight (cv. ‘Palivec’) to 259 mg N-NO_3_ kg^−1^ fresh weight (cv. ‘Turbine F_1_’).

According to Selahle et al.^35^, the taste of sweet peppers is determined by the content of sugars and organic acids. Taste is a complex phenomenon, and it is affected by environmental factors during plant growth^36,37^. From the nutritional perspective, the dry matter of pepper fruit consists of sugars, organic acids and other compounds with proven nutraceutical efficacy, including hydrophilic compounds such as ascorbic acid, flavonoids and phenolic acids, and lipophilic compounds such as carotenoids and tocopherols^38–41^. Fresh peppers are rich in valuable compounds including vitamins (in particular vitamin C), mineral salts, macronutrients and micronutrients^42^. According to Hallmann et al.^43^, pepper fruit contains on average 8.5–10.5 g 100 g fresh weight of dry matter, 3.6–6.6 g 100 g fresh weight of total sugars, 2.4–4.8 g 100 g fresh weight of reducing sugars, and 115–153 mg 100 g fresh weight of L-ascorbic acid, depending on cultivation method. Similar values were determined in the present study. The content of nitrates (V) depends on soil and climatic conditions, fertilization and plant species^44^, which were identical in all treatments in this study. The tested biostimulants exerted varied effects on the nutrient content of *C. annuum* fruit. The nitrate (V) content of fruit was higher in experimental treatments than in the control group, but the noted differences were significant only relative to treatment I where the maximum permissible level of 250 mg N-NO_3_ kg^−1^ fresh weight was exceeded^43^. ‘Turbine F_1_’, followed by ‘Solario F_1_’, were most prone to nitrate (V) accumulation in fruit. In this respect, the effect exerted by the biostimulants was undesirable.

### Correlations between the analyzed biometric parameters and chemical composition of fruit

Due to the fact that the tested biostimulants exerted no clear-cut effects on the analyzed biometric parameters of *C. annuum* fruit, and for the sake of simplicity, the measurement data were pooled into two experimental groups of sweet and hot cultivars. The results of a correlation analysis of the above parameters are presented in Table 5. The absolute values of the correlation coefficient ranged from 0.012 (correlation between the L-ascorbic acid content and nitrate (V) content of fruit in sweet cultivars) to 0.932 (correlation between the weight and horizontal diameter of fruit in sweet cultivars). Significant correlations were noted in 36 cases out of 72 comparisons, whereas practical significance (coefficient of correlation minimum 0.4) was observed in 33 comparisons. The nitrate (V) content of fruit was least frequently correlated, and the horizontal diameter, total sugar content and reducing sugar content of fruit were most frequently correlated with the remaining parameters. The nature of relationships between the analyzed parameters was largely affected by the type of cultivar. Differences in the significance of correlation coefficients were found in 19 pairs of the compared traits, and differences in their direction (positive, negative) were observed in 11 pairs out of 36 comparisons. The significance and direction of correlations were consistent only with regard to horizontal diameter vs. the total sugar content and reducing sugar content, and skin thickness vs. reducing sugar content and L-ascorbic acid content. This implies that irrespective of cultivar, an increase in the horizontal diameter of fruit was associated with an increase in sugar content, and an increase in skin thickness was associated with an increase in the content of reducing sugars and L-ascorbic acid. Therefore, it can be assumed that the fruits characterized by a larger horizontal diameter and thicker skin are richer in nutrients.

**Table 5:** Pearson’s coefficients of correlation between the analyzed parameters of *Capsicum annuum* fruit.

Type of cultivar	Parameter	*m*_*f*_	*d*_1_	*d*_2_	*s*	*c*_*dm*_	*c*_*ts*_	*c*_*rs*_	*c*_*L*_
Sweet	*d*_1_	0.225	1						
	*d*_2_	**0.932**	0.229	1					
	*s*	0.083	**0.498**	0.022	1				
	*c*_*dm*_	**0.632**	**0.371**	**0.665**	0.161	1			
	*c*_*ts*_	**0.583**	0.192	**0.557**	0.226	0.289	1		
	*c*_*rs*_	**0.530**	**0.347**	**0.429**	**0.518**	0.221	**0.850**	1	
	*c*_*L*_	0.286	**0.657**	**0.360**	**0.538**	**0.439**	0.189	0.179	1
	*c*_*N*_	0.286	0.174	0.194	− 0.067	**0.456**	− 0.015	0.090	− 0.012
Hot	*d*_1_	**0.823**	1						
	*d*_2_	− **0.506**	− **0.600**	1					
	*s*	0.122	− 0.180	**0.650**	1				
	*c*_*dm*_	0.279	0.288	0.255	0.070	1			
	*c*_*ts*_	− 0.141	− **0.423**	**0.746**	**0.588**	**0.545**	1		
	*c*_*rs*_	− **0.425**	− **0.711**	**0.740**	**0.632**	− 0.266	**0.483**	1	
	*c*_*L*_	**0.584**	0.283	0.371	**0.796**	0.360	**0.506**	0.274	1
	*c*_*N*_	0.185	− 0.388	**0.684**	0.372	**0.537**	**0.768**	**0.533**	0.262

*m*_*f*_-weight, *d*_1_-vertical diameter, *d*_2_-horizontal diameter, *s*-skin thickness, *c*_*dm*_-dry matter content, *c*_*ts*_-total sugar content, *c*_*rs*_-reducing sugar content, *c*_*L*_-L-ascorbic acid content, *c*_*N*_-nitrate (V) content.

Bold values denote significant correlations at 0.05.

**Figure 1:**
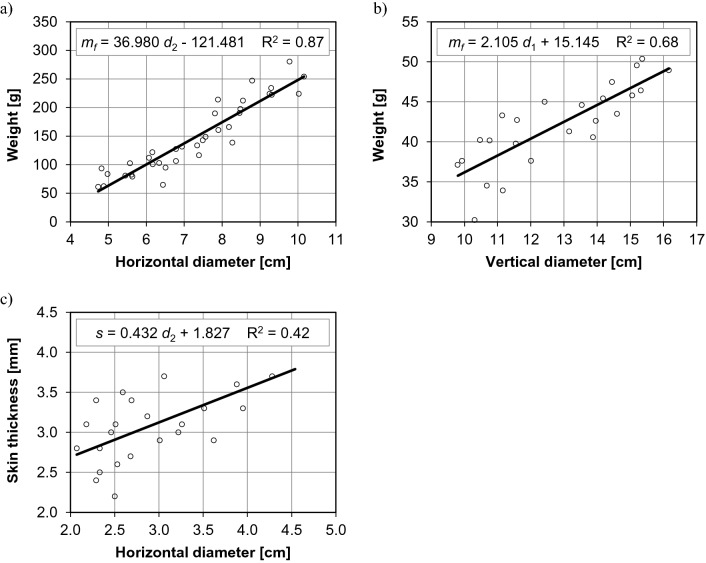
Relationships between the biometric parameters of *Capsicum annuum* fruit: (**a**) horizontal diameter and weight of sweet peppers, (**b**) vertical diameter and weight of hot peppers, (**c**) horizontal diameter and skin thickness of hot peppers.

In the group of fruit biometric parameters, the strongest correlation was found between the horizontal diameter and weight of fruit in sweet cultivars (coefficient of determination R^2^ = 0.87), and it was well described by a linear function (Fig. 1a). An increase in the horizontal diameter of fruit from around 4.7 cm to around 10.2 cm was accompanied by a proportional increase in fruit weight from around 53 g to around 254 g (by approx. 380%). Equations with the minimum value of the determination coefficient (0.4) were also derived for the correlations between the vertical diameter and weight of fruit, and between the horizontal diameter and skin thickness of fruit in hot cultivars (Figs. 1b and 1c). An increase in the vertical diameter of fruit by around 65% increased their weight by around 40%, and an increase in the horizontal diameter of fruit by around 120% increased their skin thickness by around 40%.

**Figure 2:**
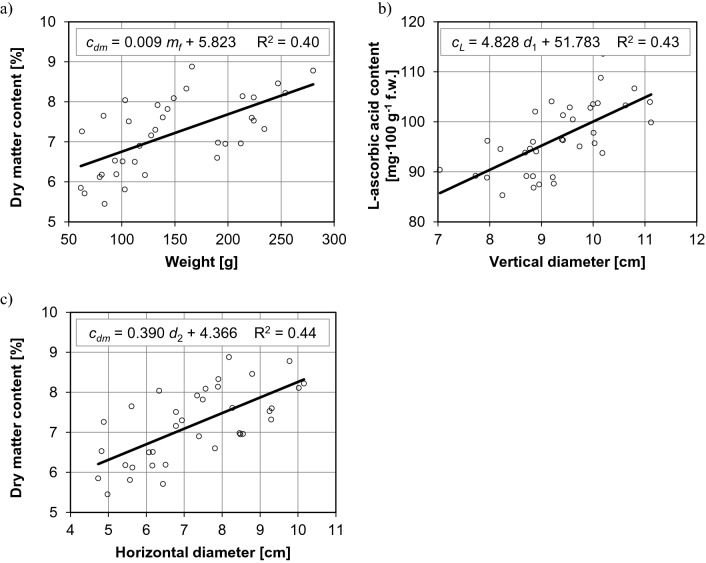
Relationships between the biometric parameters and chemical composition of fruit in sweet cultivars of *Capsicum annuum*: (**a**) weight and dry matter content, (**b**) vertical diameter and L-ascorbic acid content, (**c**) horizontal diameter and dry matter content.

**Figure 3:**
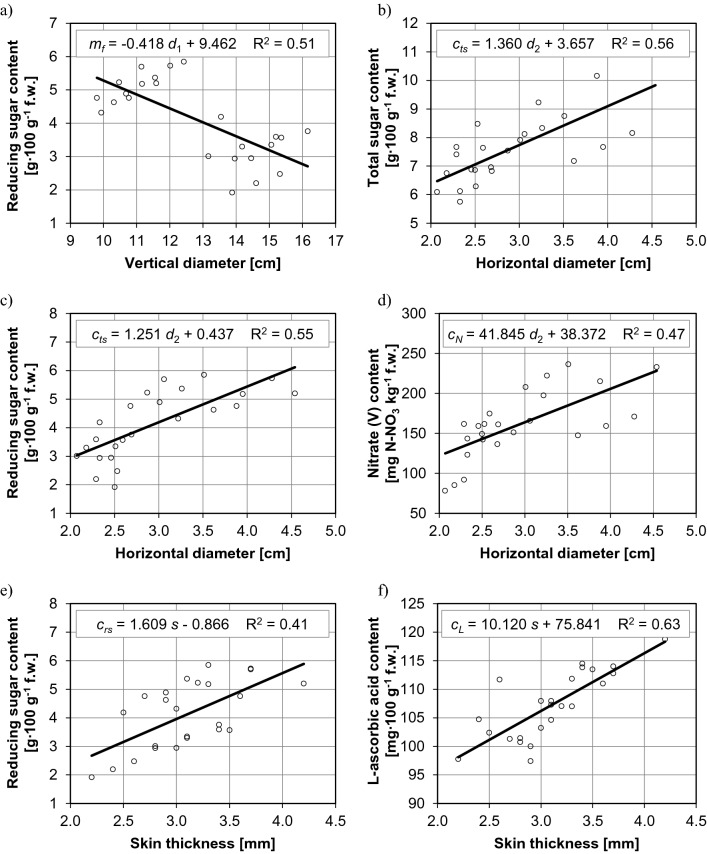
Relationships between the biometric parameters and chemical composition of fruit in hot cultivars of *Capsicum annuum*: (**a**) vertical diameter and reducing sugar content, (**b**) horizontal diameter and total sugar content, (**c**) horizontal diameter and reducing sugar content, (**d**) horizontal diameter and nitrate (V) content, (**e**) skin thickness and reducing sugar content, (**f**) skin thickness and L-ascorbic acid content.

The biometric parameters and chemical composition of pepper fruit are strongly correlated (Figs. 2 and 3). Three and six equations with the minimum value of the determination coefficient (0.4) were derived for the correlations between fruit parameters in sweet and hot pepper cultivars, respectively. In sweet cultivars, the dry matter content of fruit was affected by their weight and horizontal diameter, and the noted relationships were directly proportional. An increase in fruit weight by around 360% (Fig. 2a) and an increase in the horizontal diameter of fruit by around 115% (Fig. 2c) increased their dry matter content by around 35%. An increase in the vertical diameter of fruit by around 60% increased their L-ascorbic acid content by around 25% (Fig. 2b). In hot cultivars, the chemical composition of fruit was most significantly influenced by horizontal diameter, followed by skin thickness. An increase in the horizontal diameter of fruit from around 2.0 cm to around 4.5 cm was accompanied by an increase in their total sugar content by around 50% (from approx. 6.5 g∙100 g^−1^ fresh weight to approx. 9.8 g∙100 g^−1^ fresh weight) (Fig. 3b), reducing sugar content—by around 100% (from approx. 3.0 g∙100 g^−1^ fresh weight to approx. 6.1 g∙100 g^−1^ fresh weight) (Fig. 3c) and nitrate (V) content—by around 80% (from approx. 125 mg N-NO_3_ kg^−1^ fresh weight to approx. 228 mg N-NO_3_ kg^−1^ fresh weight) (Fig. 3d). In turn, an increase in skin thickness (from approx. 2.2 mm to approx. 4.2 mm, by approx. 90%) was accompanied by an increase in reducing sugar content (Fig. 3e) and L-ascorbic acid content (from approx. 98 mg∙100 g^−1^ fresh weight to approx. 118 mg∙100 g^−1^ fresh weight, by approx. 20%) (Fig. 3f). An increase in the vertical diameter of fruit from around 9.8 cm to around 16.2 cm decreased their reducing sugar content by around 50% (Fig. 3a).

**Figure 4:**
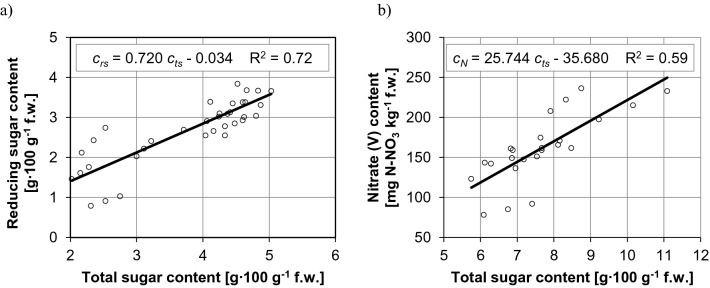
Relationships between the chemical composition of *Capsicum annuum* fruit: (**a**) total sugar content and reducing sugar content of sweet peppers, (**b**) total sugar content and nitrate (V) content of hot peppers.

In the group of the chemical composition parameters of fruit, the strongest correlation was found between total sugar content and reducing sugar content (R^2^ = 0.72) in sweet cultivars (Fig. 4a), and between total sugar content and nitrate (V) content (R^2^ = 0.59) in hot cultivars (Fig. 4b). These relationships can be described by linear functions. An increase in the total sugar content of sweet peppers from around 2.0 g∙100 g^−1^ fresh weight to around 5.0 g∙100 g^−1^ fresh weight (by approx. 150%) was accompanied by an increase in reducing sugar content by around 150%, which indicates that the ratio between both sugar fractions remained unchanged. An increase in the total sugar content of hot peppers from around 5.8 g∙100 g^−1^ fresh weight to around 11.1 g∙100 g^−1^ fresh weight (by approx. 90%) was accompanied by an increase in nitrate (V) content from around 110 mg N-NO_3_ kg^−1^ fresh weight to around 250 mg N-NO_3_ kg^−1^ fresh weight (by approx. 120%).

## Materials and methods

### Study sites and experimental factors

A two-factorial experiment was established in 2017 and 2018 in the Agricultural Experiment Station owned by the University of Warmia and Mazury (UWM) in Olsztyn, Faculty of Agriculture and Forestry (53.753569°N and 20.455521°E). *Capsicum annuum* plants were grown in an unheated plastic tunnel. The experiment was carried out in accordance with the "Methodology of Integrated Peppers Production"^6^.

The first experimental factor (A) were three sweet cultivars- ‘Solario F_1_’, ‘Turbine F_1_’ and ‘Whitney F_1_’, and two hot cultivars—‘Cyklon’ and ‘Palivec’ of *C. annuum*. The second experimental factor (B) was the effect exerted by BB Soil, BB Foliar, Multical, MK5 and Biocin F biostmulants on the growth, development and yield of peppers. The biostimulants were applied at regular intervals, as recommended by the supplier, Agrosystemy Ltd., in the following single doses^22^:

- BB Soil (2 l·ha^−1^),
- BB Foliar (1.5 l·ha^−1^),
- Multical (1.5 kg·ha^−1^),
- MK5 (0.5 l·ha^−1^),
- Biocin F (2 l·ha^−1^).

In the control treatment, the plants were not sprayed with the tested biostimulants.

### Cultivation in a plastic tunnel

Seedlings were grown in pots in a heated greenhouse. Before the experiment, chemical analyses of the substrate were performed. The content of N-NO_3_ was determined by the colorimetric method with the use of phenol disulfonic acid (UV-1201 V spectrophotometer, Shimadzu Corporation Kyoto, Japan). Phosphorus content was determined by the vanadium molybdate yellow colorimetric method (UV-1201 V spectrophotometer, Shimadzu Corporation Kyoto, Japan), and the content of calcium and potassium was determined by atomic emission spectrometry (AES) (BWB Technologies UK Ltd. Flame Photometers). Magnesium was extracted with 0.01 M CaCl_2_ and its content was determined by atomic absorption spectrophotometry (AAS) (AAS1N, Carl Zeiss Jena, Germany). Salinity was determined by the conductometric method (N5773 conductivity meter, Teleko Wrocław, Poland), and pH in H_2_O was determined by the potentiometric method. Containers with bottomless rings were filled with sphagnum peat with salt concentration of 1.5 g·dm^−3^, pH 4.99 and the following chemical composition: N-NO_3_-112 mg·dm^−3^, P-257 mg·dm^−3^, K-1433 mg·dm^−3^, Ca-1050 mg·dm^−3^, Mg-383 mg·dm^−3^.

Pepper seeds were sown in the first week of March. Mean air temperature and humidity in the greenhouse were 24–26 °C and 80%, respectively. Microclimate was controlled automatically with the use of a climate control computer. After emergence, mean air temperature and humidity in the greenhouse were 20–21 °C and 60%, respectively. After 14 days, the seedlings were planted out into 7 dm^3^ bottomless rings filled with the substrate. Initially, the pots were set out in one block. At a later growth stage, the pots were arranged in chessboard fashion at a spacing of 0.1 × 0.2 m to prevent contact between leaves. Beginning on the 1^st^ of May, the seedlings were hardened off by lowering the temperature to 18–19 °C during the day and at night, and restricted watering. When the first symptoms of Crescent-marked lily aphid infestation were observed, the seedlings were sprayed with Kohinor 200SL (ADAMA Poland Ltd., Warsaw) at a concentration of 0.4%. The first buds were removed to strengthen the plants.

Thirty days before the experiment, the tunnel (30 × 6 m) was covered with plastic sheeting, and soil was prepared with a rotary cultivator (two passes at a depth of 30 cm and speed of approx. 0.1–0.3 m·s^−1^). Seven days before transplanting, 7 dm^3^ rings filled with standard substrate was arranged at a spacing of 0.4 × 0.5 m, in four double rows. The total area under cultivation was 90 m^2^. Watering hoses with emitters were placed in each row, with one pressure-compensating emitter per ring. The seedlings were transplanted in the first week of May.

In all treatments, plants were fertilized in accordance with the recommendations for *C. annuum*^6,22^. Once a week (Monday, 8.00–9.00 a.m.), a solution of BB Soil at 2 l·ha^−1^ was applied to the leaves of plants in all treatments except the control. BB Soil contains a mixture of lactic acid bacteria (*Lactobacillus casei*, *Lactobacillus plantarum*), photosynthetic bacteria (*Rhodopseudomonas palustris*) and yeasts (*Saccharomyces cerevisiae*). It activates soil life, contributes to more rapid warming of the soil in spring, and increases the water retention capacity of soil. It also stimulates plant growth and enhances plant resistance^22^. Three times a week (Monday, Wednesday and Friday, 3.00–4.00 p.m.), a solution of BB Soil at 2 l·ha^−1^ was also applied directly to the rings in all treatments except the control where the plants were only watered six times. The EC of the solution was 2.2–2.5 mS·cm^−1^, and its pH was 6.0–6.5. The solution was diluted (1:250) before application. In order to prepare a smaller amount of working solution, the recommended dose was adjusted proportionally. In addition, biostimulants were applied to plants every seven days (Monday, 8.00–9.00 a.m.), according to the following design:

- treatment C (control)-no biostimulant or BB Soil sprays;
- treatment I-BB Foliar applied at 1.5 l·ha^−1^ and Multical applied at 1.5 kg·ha^−1^;
- treatment II-BB Foliar applied at 1.5 l·ha^−1^, Multical applied at 1.5 kg·ha^−1^ and MK5 applied at 0.5 l·ha^−1^;
- treatment III-BB Foliar applied at 1.5 l·ha^−1^, Multical applied at 1.5 kg·ha^−1^, MK5 applied at 0.5 l·ha^−1^ and Biocin F applied at 2 l·ha^−1^.

The above biostimulants are biological, environmentally-friendly formulations that support plant growth and development. BB Foliar is a microbial solution that consists mainly of lactic acid bacteria, photosynthetic bacteria and yeasts, and it contains no genetically modified microorganisms. Multical is a plant-strengthening product. It is authorized in organic farming, and poses no threat to humans, animals, groundwater or protected areas. Multical optimizes nutrient absorption, promotes rapid plant growth and abundant flowering. It contains no genetically modified microorganisms. This solution provides systemic protection against pests and fungi. It contains 100% natural plant-derived trace minerals that are readily available for plant uptake. MK5 contains a mixture of lactic acid bacteria (*Lactobacillus casei, Lactobacillus plantarum*), photosynthetic bacteria (*Rhodopseudo-monas palustris*) and yeasts (*Saccharomyces cerevisiae*), as well as sugarcane molasses, fermented vinegar, alcohol, water, garlic chili pods. The product contains no genetically modified microorganisms, and it is used in many countries as a natural plant protection agent. It is a dark-brown, sweetly acidic liquid. Biocin F is composed of 100% natural plant-derived trace minerals that are readily available for plant uptake. According to the manufacturer^16^, it contains K-200 m L^−1^, B-0.1 m L^−1^, S-0.01 m L^−1^, Ca-85 m L^−1^, Mg-13 m L^−1^ and Si-22 m L^−1^.

In each treatment, peppers of five cultivars (12 plants per cultivar) were grown in two belts. The plants were watered on a regular basis, and weeds were removed manually.

### Biometric parameters of plants and fruit

On day 15 of each month, leaf greenness was measured with the SPAD-502 chlorophyll meter (Konica Minolta INC, Wrocław, Poland). The measurements were performed on three youngest fully developed leaves of five plants selected randomly in each treatment, and the results were averaged. At the end of the experiment, the weights of aboveground parts and roots of each plant were determined to the nearest 1 g using the Radwag PST 750 R2 laboratory precision balance (Radwag, Radom, Poland).

Pepper fruits were harvested gradually upon reaching maturity, once a week, on Friday morning, in accordance with Commission Regulation (EC) No. 1455/1999 of 1 July 1999 as last amended by Regulations (EC) No. 2706/2000 and No. 46/2003^21^. In all treatments, ripening fruits were first observed in plants of cv. ‘Whitney F_1_’, whereas ‘Solario F_1_’ was the latest-ripening cultivar.

The early yield (the first three harvests), total yield (comprising all fruits that developed on plants) and marketable yield (comprising healthy and well-developed fruits) of *C. annuum*, and the biometric parameters of fruit were determined. At harvest, in the last week of July, the biometric parameters of plants were also measured with a caliper (accurate to ± 1 mm) and a ruler (accurate to ± 1 mm). Ten fruits were collected from plants in each plot to determine their weight, vertical diameter (length) and horizontal diameter (width), and pericarp thickness.

### Chemical analysis of fruit

The chemical composition of fruit was determined in the first week of August. Fruits were collected from the marketable yield in each replicate, to prepare the average sample per treatment. Pepper fruits were analyzed in the laboratory of the Department of Horticulture, UWM in Olsztyn to determine:

- dry matter-by drying to constant weight at 105 °C in the Pol-Eko Aparatura SLW 535 SD laboratory dryer (Pol-Eko, Wodzisław Śląski, Poland)^46^;
- total and reducing sugars in fresh weight-by the Luff-Schoorl method^47^;
- L-ascorbic acid in fresh weight-by the method proposed by Tillmans and modified by Pijanowski^48^;
- nitrates (V) content-by the colorimetric method with the TECHCOMP UV2310II spectrometer (Techcomp, Ltd., Shanghai, China), using salicylic acid^49^.

### Statistical analysis

The experiment was conducted under identical, controlled conditions over two consecutive years (2017 and 2018), therefore the results were presented as two-year means. The results of measurements of the physical parameters and chemical composition of pepper fruit were analyzed statistically using Statistica Pl ver. 13.3 software (TIBCO, Paolo Alto, CA); the results were regarded as statistically significant at α = 0.05. The variations in the analyzed traits were determined by two-way analysis of variance (ANOVA). Homogeneous groups were identified by Tukey’s test. The strength of relationships between the physical properties of plants and fruits was evaluated by calculating Pearson’s coefficients of correlation, and the functions describing those relationships were derived by regression analysis. The functions available in Statistica were tested, and the simplest function with a sufficiently high coefficient of determination (minimum 0.4) was selected.

## Conclusions

The application of biostimulants in *C. annuum* cultivation contributed to an increase in the nitrate (V) content of fruit, which is undesirable from a consumer’s perspective. The fruits of sweet cultivars, in particular cvs. Turbine F_1_’ and ‘Solario F_1_’, were most prone to nitrate (V) accumulation in fruit, and the fruits of the hot cultivar ‘Palivec’ had the lowest nitrate (V) content. In view of the above, treatment II (BB Soil, BB Foliar, Multical and MK5) was optimal. The increase in fruit yield, noted in treatment II (early yield—by approx. 20%, marketable and total yields—by approx. 15%, compared with the control treatment), resulted mainly from the relatively high weight of fruit. However, the observed differences were not significant. Therefore, the measurable benefits of biostimulants in *C. annuum* cultivation do not balance the costs of their purchase and application. The biometric parameters and nutrient content of pepper fruits did not differ between the treatments where biostimulants were and were not applied.

Sweet pepper cultivars, compared with hot cultivars, were characterized by higher fruit yield, but a higher percentage of fruits were unsuitable for sale. The weight of sweet peppers is significantly correlated with their horizontal diameter, and the weight of hot peppers—with their vertical diameter. The horizontal and vertical diameters can be used as separation traits for calibrating and sorting pepper fruits intended for sale.

Pepper fruits with a larger horizontal diameter and thicker skin should be preferred due to their higher nutrient (sugar) content.
