# Author Correction: Effect of biostimulants on the growth, yield and nutritional value of *Capsicum annuum* grown in an unheated plastic tunnel

**DOI:** 10.1038/s41598-021-03464-9

**Published:** 2021-12-08

**Authors:** Joanna Majkowska-Gadomska, Artur Dobrowolski, Krzysztof K. Jadwisieńczak, Zdzisław Kaliniewicz, Anna Francke

**Affiliations:** 1 Department of Agroecosystems and Horticulture, Faculty of Agriculture and Forestry, University of Warmia and Mazury in Olsztyn, Plac Łódzki 3, 10–718, Olsztyn, Poland; 2 Department of Heavy Duty Machines and Research Methodology, University of Warmia and Mazury in Olsztyn, Oczapowskiego 11, 10–719, Olsztyn, Poland

Correction to: *Scientific Reports* 10.1038/s41598-021-01834-x, published online 16 November 2021

The Funding section in the original version of this Article was incorrect.

“The results presented in this paper were obtained as part of a comprehensive study financed by the University of Warmia and Mazury in Olsztyn, Faculty of Agriculture and Forestry, Department of Agroecosystems and Horticulture, 30.610.016–110. Project financially supported by Minister of Education and Science in the range of the program entitled “Regional Initiative of Excellence” for the years 2019–2022, Project No. 010/RID/2018/19.”

now reads:

“The results presented in this paper were obtained as part of a comprehensive study financed by the University of Warmia and Mazury in Olsztyn, Faculty of Agriculture and Forestry, Department of Agroecosystems and Horticulture, 30.610.016-110. Project financially supported by Minister of Education and Science in the range of the program entitled “Regional Initiative of Excellence” for the years 2019–2022, Project No. 010/RID/2018/19, amount of funding 12.000.000 PLN.”

The original Article has been corrected.

